# Identification of data elements for blood gas analysis dataset: a base for developing registries and artificial intelligence-based systems

**DOI:** 10.1186/s12913-022-07706-y

**Published:** 2022-03-08

**Authors:** Sahar Zare, Zahra Meidani, Maryam Ouhadian, Hosein Akbari, Farid Zand, Esmaeil Fakharian, Roxana Sharifian

**Affiliations:** 1 Health Information Management Research Center (HIMRC), Kashan University of Medical Sciences, Kashan, Iran; 2 Department of Health Information Management & Technology, School of Allied Health Professions, Kashan University of Medical Sciences, Kashan, Iran; 3 Anesthesiology and Critical Care Research Center, Shiraz University of Medical Sciences, Shiraz, Iran; 4 Department of Epidemiology and Biostatistics, School of Health, Kashan University of Medical Sciences, Kashan, Iran; 5 Department of Anesthesia and Critical Care Medicine, Shiraz University of Medical Sciences, Shiraz, Iran; 6 Department of Neurosurgery, Trauma Research Center, Kashan University of Medical Sciences, Kashan, Iran; 7 Health Human Resources Research Center, Department of Health Information Management and Technology, Shiraz University of Medical Sciences, Shiraz, Iran

**Keywords:** Blood gas analysis, Databases, Information science, Artificial intelligence, Clinical decision-making

## Abstract

**Background:**

One of the challenging decision-making tasks in healthcare centers is the interpretation of blood gas tests. One of the most effective assisting approaches for the interpretation of blood gas analysis (BGA) can be artificial intelligence (AI)-based decision support systems. A primary step to develop intelligent systems is to determine information requirements and automated data input for the secondary analyses. Datasets can help the automated data input from dispersed information systems. Therefore, the current study aimed to identify the data elements required for supporting BGA as a dataset.

**Materials and methods:**

This cross-sectional descriptive study was conducted in Nemazee Hospital, Shiraz, Iran. A combination of literature review, experts’ consensus, and the Delphi technique was used to develop the dataset. A review of the literature was performed on electronic databases to find the dataset for BGA. An expert panel was formed to discuss on, add, or remove the data elements extracted through searching the literature. Delphi technique was used to reach consensus and validate the draft dataset.

**Results:**

The data elements of the BGA dataset were categorized into ten categories, namely personal information, admission details, present illnesses, past medical history, social status, physical examination, paraclinical investigation, blood gas parameter, sequential organ failure assessment (SOFA) score, and sampling technique errors. Overall, 313 data elements, including 172 mandatory and 141 optional data elements were confirmed by the experts for being included in the dataset.

**Conclusions:**

We proposed a dataset as a base for registries and AI-based systems to assist BGA. It helps the storage of accurate and comprehensive data, as well as integrating them with other information systems. As a result, high-quality care is provided and clinical decision-making is improved.

## Background

Artificial intelligence (AI) has revolutionized the health care industry. The AI technologies allow data analysts to transform raw data generated in healthcare facilities into meaningful insights for an effective decision-making process [1]. The large amount of data generated daily in health facilities makes decision-making difficult. Clinical decision support systems are a subset of AI designed to facilitate decision-making in healthcare facilities using a large amount of data, medical knowledge, and analysis engines. These systems make patient-specific assessments or recommendations for healthcare providers [2].

One of the challenging decision-making tasks in healthcare centers is the interpretation of blood gas tests. Arterial/venous blood gas tests are among the high-cost and frequently-ordered tests in intensive care units (ICUs). These tests demonstrate the respiratory and metabolic status of patients, as well as acid–base balance [3, 4]. Acid–base imbalance can cause negative outcomes in patients, such as damage to the kidneys, cardiovascular system, and nervous system; if serious, it can be considered as a risk factor for death [5]. Consequently, the rapid diagnosis of blood gas disorders and acid–base imbalance can prevent severe complications. In order to make these tests effective diagnostic tools, physicians need to be professional in interpreting blood gas analysis (BGA). However, in contrast to other tests with values higher or lower than normal, BGA contains more than six parameters, which are complicated and difficult to interpret [6].

To simplify the interpretation of BGA, AI-based decision support systems can be highly useful [7]. These systems assist healthcare providers by transforming raw health data, documents, and expert practice into sophisticated algorithms or techniques, such as machine learning or knowledge graphs. As a result, healthcare decision-makers can find appropriate solutions to the underlying medical problems [8]. AI-based decision support systems can support BGA according to their knowledge base and predefined algorithms.

An initial step for developing intelligent systems is to determine information requirements and automated input of data for secondary analyses [9]. Jamieson et al [10]. found that electronic documentation improves the quality of documentation. The interoperability of data among information systems is necessary for the automatic input of data. Datasets can help automated input of data from dispersed information systems [11, 12]. Dataset is a comprehensive data element list on a specific clinical condition [13], procedure [14], specialty [15], healthcare process [16], or an entire domain with broad scope [17].

Datasets may include historical data which can help us interpret an impression, a diagnosis, or a treatment for planning future follow-up strategies [9]. In order to develop a robust AI-based system, one should ensure seamless and comprehensive access to the related information, suggestively an integrated data view comprising of electronic health records, computerized physician order entry, laboratory systems, and other related applications. Such an arrangement would facilitate access to information as a comprehensive centralized data repository, which can be used to support various clinical decision support systems, machine learning, data mining, and deep learning. Moreover, the quality of data remarkably affects the standards and outcomes of the resultant decision support system [18]. The quality of data can be enhanced by proper structuring following the data standardization approach [19]. Datasets have been used in previous researches for AI-based technologies, including machine learning, deep learning, and data mining. For instance, Muhammad et al*.* applied machine learning models for the prediction of Coronavirus disease 2019 using an epidemiology dataset [20]. Hussain et al*.* also applied data mining algorithms on an accident dataset to determine the causes of accidents or prone locations [21].

Langarizadeh and Gholinezhad [22] have emphasized the role of defining datasets in laboratory reports, such as demographic, administrative, clinical, insurance, anesthesia, laboratory, observation, and interpretation for exchanging with information systems. A blood gas test needs a dataset as a base for developing AI-based systems. To our knowledge, there is no dataset developed for BGA. Therefore, the present study aimed to identify the data elements required for supporting BGA as a dataset.

## Materials and methods

### Study design and setting

This cross-sectional descriptive study was conducted in 2020–2021. Experts from two hospitals affiliated to Shiraz University of Medical Sciences, namely Nemazee and Rajaee hospitals, in addition to experts from Kashan University of Medical Sciences participated in this study. The present study was conducted in Nemazee Hospital with 925 active beds as the largest educational and treatment center in Shiraz and the only referral hospital in Southern Iran. This hospital is also a pioneer in developing information systems, especially for ICUs [23, 24].

### Data elements identification

A combination of literature review, experts’ consensus, and the Delphi technique was used to identify the data elements.

### Stage one: literature review

To determine the data elements for the BGA dataset, first, a review of the literature was performed on the electronic databases of Cochrane Library, PubMed, and SCOPUS. A combination of terms related to dataset or registry (e.g., “dataset”, OR “common data”, OR “element”, OR “MDS”, OR “algorithms”, OR “Guideline”, OR “Clinical Protocols”, OR “registries”, “information system”, OR “electronic health record”, OR “database” AND terms related to blood gas, including “Blood Gas Analysis”, OR “arterial blood gas”, OR “venous blood gas”, OR “ABG”, OR “VBG” were searched in titles and abstracts were performed. In addition, a manual search of the related textbooks, patients’ records, and the following websites was performed: “American Thoracic Society”, “American Association for Critical Care Nurses”, “Respirology”, “European Respiratory Society”, “British Association for Critical Care Nurses”, and “Emergency Medical Journal and Thorax”.

### Inclusion and exclusion criteria

Any relevant papers reporting the indications or considerations for ordering BGA, as well as papers reporting any influential factors in BGA, or presenting a protocol, algorithm, rules, or explanation on how to analyze the blood gas results were included. Moreover, the existing datasets or registries capturing the data related to blood gas disorders were investigated [25–27]. Any report, guideline, and form available on the searched websites were also included. Studies were included without time limit if were published in the English language and their full text contained the determined keywords in the title or abstracts. Single case reports and studies on neonates, children, or animals were excluded.

### Stage two: experts’ consensus

A team of four experts, including a critical care specialist, a general practitioner with sufficient knowledge about blood gases, and two health information management specialists, was formed as an expert panel. The list of data elements extracted through a literature search was presented to the expert panel. Several sessions were held to tailor the initial draft of the dataset to the specific needs and practices of the ICUs by incorporating the opinion of medical specialists. Experts were invited to discuss on, add, or remove the data elements presented in the draft dataset. The criteria that might influence blood gas based on rational principles and are likely to be considered by physicians when interpreting the test results or are used for taking actions received higher scores. On the other hand, the criteria that do not affect blood gas received lower scores.

Eleven expert panel sessions were held to finalize the dataset. These expert panels started on 10 November 2020 and ended on 2 May 2021. Some of these sessions were held in the office of central ICU in Nemazee Hospital and some were held in the Anesthesiology and Critical Care Research Center affiliated to Shiraz University of Medical Sciences. Diseases in the draft dataset were categorized based on the eleventh version of the International Statistical Classification of Diseases and Related Health Problems (ICD-11). After finalizing the initial draft of the dataset in expert panel sessions, the dataset was presented as a checklist, the content validity of which was confirmed by four experts, including two other critical care specialists, one internal medicine specialist, and one health information management. They assessed the criteria in terms of clarity, contribution to BGA, and interpretability.

### Stage three: delphi technique

Delphi technique was used to reach consensus and validate the draft dataset. Delphi technique is utilized by researchers when the available knowledge/information/dataset/study is incomplete or is subjected to uncertainty and hence, a group opinion or decision is made based on the interaction between the researchers and a group of identified experts [28]. Another group of experts, including two anesthesiologists, two critical care specialists, two nephrologists, and two neurosurgeons were invited to review the dataset draft. The researcher presented the questionnaire to the experts and a face-to-face brief explanation was given about the study and the dataset design. These experts were asked to answer the questionnaire based on “Yes” (including mandatory and optional) and “No” options. Mandatory or optional were selected based on the impact of the data element on BGA or the complication of the results, as well as their prevalence/frequency of use (for diseases, medications, or toxins). Furthermore, “mandatory” data elements are those required when the user expects AI-based decision support systems to present a simple BGA. On the other hand, “optional” data elements are those needed when the user expects an advanced comprehensive BGA. Previous studies mostly focused on simple BGA [6, 29, 30]. However, in the current study, we created the "mandatory" and "optional" divisions to determine data elements required for simple and advanced BGA, respectively. A blank row was considered at the end of each section for experts to leave comments or to add necessary data elements. If 75% or more experts selected the “YES” option (either mandatory or optional), the data element was considered to be contained in the datasets. If 50% of experts selected the “NO” option, the data element was removed. If the consensus was between 50%-75%, the data elements needed revision. Six anesthesiologists and critical care attendants participated in another expert panel to discuss on and decide about the inclusion or exclusion of data elements with a 50%-75% consensus. The reliability of the dataset was evaluated based on the split-half method (the Guttman split-half coefficient was 0.83).

## Results

**Fig. 1:**
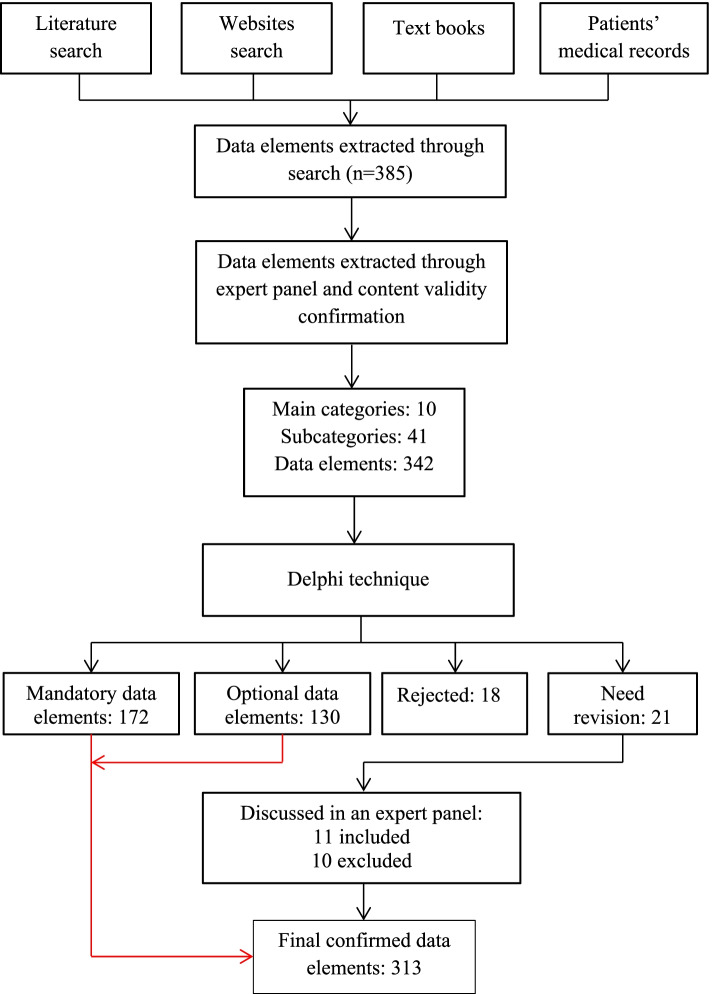
Flowchart of data elements determination

As shown in Fig. 1, following the literature review step, 385 data elements were extracted. After expert panel sessions, 43 data elements were deemed unnecessary and were excluded. Delphi technique also resulted in the exclusion of 18 data elements. Moreover, 21 data elements obtained a consensus rate of 50%-75% and needed revision. An expert panel was held to discuss the latter 21 data elements, of which 11 were excluded resulting in 313 data elements. Table 1 shows the agreement level between Delphi method and the experts voting in each level.

**Table 1:** Agreement levels in Delphi method and the experts voting in each level

Agreement level	Decision on the data element	Percentage of experts voting
≥ 75%	Accepted	88.30%
50 < agreement < 75	Discussed on the expert panel	6.14%
≤ 50%	Declined	5.26%

The dataset of BGA was categorized into ten categories: 1) Personal information, 2) Admission details, 3) Present illnesses, 4) Past medical history, 5) Social status, 6) Physical examination, 7) Paraclinical investigation, 8) Blood gas parameter, 9) Sequential organ failure assessment (SOFA) score, and 10) Sampling technique errors (ABG Error). Overall, 313 data elements, including 172 mandatory and 141 optional data elements were confirmed by the experts to be contained in the dataset (Table 2).

**Table 2:** The categories and subcategories of the proposed dataset

**Category**	**Subcategory**	**Number of data elements**	**Mandatory**	**Optional**
**1-Personal Information**	1–1-Personal Information	**10**	**10**	**0**
**2-Admission Details**	2–1-Admission Details	**10**	**9**	**1**
**3-Present illness**	3–1-Respiratory disease	10	9	1
	3–2-Renal disease	6	3	3
	3–3-Gastrointestinal disease/ Liver disease	6	6	0
	3–4-Endocrine disease	7	2	5
	3–5-Cardiovascular disease	3	3	0
	3–6-Hematologic disease	2	0	2
	3–7-Neurologic disease	6	5	1
	3–8-Infectious disease	3	1	2
	3–9-Trauma	6	3	3
	3–10-Drugs	54	33	21
	3–11-Toxins	21	5	16
	Total	125	71	54
**4-Past Medical History:**	4–1-Respiratory disease	9	5	4
	4–2-Renal disease	18	8	10
	4–3-Gastrointestinal disease/ Liver disease	7	2	5
	4–4-Endocrine disease	24	3	21
	4–5-Cardiovascular disease	1	1	0
	4–6-Hematologic disease	5	1	4
	4–7-Neurologic disease	5	4	1
	4–8-Genetic/Congenital disorders	18	0	18
	4–9-Rheumatology/ musculoskeletal disease	4	0	4
	4–10- Malignancy	1	0	1
	Total	92	24	68
**5-Social status**	5–1-Social status	4	4	0
**-Physical examination**	6–1-vital signs	7	7	0
	6–2-GCS (physician note)	1	1	0
	6–3-Respiratory (FiO2%):	4	4	0
	6–4-Sedation status (RAS score)	1	1	0
	6–5-Numeric pain scale	1	1	0
	6–6-Behavioral Pain Score	1	1	0
	6–7-Diaphoresis	1	1	0
	6–8-Shivering	1	1	0
	6–9-Cyanosis (if spO2 unavailable or suspicious)	1	1	0
	6–10-Urine output	1	1	0
	6–11-Nasogastric drainage	1	1	0
	6–12-Edematous states	1	1	0
	6–13-Poor tissue perfusion (regional hypo-perfusion)	1	1	0
	Total	22	22	0
**7-Para-clinical investigation**	7–1- Para-clinical investigation	29	11	18
**8-Blood gas parameter**	8–1- Blood gas parameter	7	7	0
**9-SOFA score**	9–1- SOFA score	6	6	0
**10-Sampling technique**	10–1- Sampling technique errors (ABG Error)	9	9	0
	Total	313	172	141

Essential data elements of “personal information” entailed medical record number, national code, first and last name, father's name, age, gender, birth date, estimated height, and estimated weight. “Admission details” include date/time of admission to hospital/ICU, admission type, surgical admission, insurance coverage, primary diagnosis, ICU diagnosis, and ICU intervention.

“Present illnesses” were defined as diseases that influence BGA and affected patients during the week before admission to the hospital. “Present illnesses” and “Past medical history” both included the subcategories of respiratory disease, renal disease, gastrointestinal disease/ liver disease, endocrine disease, cardiovascular disease, hematologic disease, and neurologic disease. However, the subcategories did not contain the same data elements. In addition, “Present illnesses” included infectious disease, trauma, drugs, and toxins as the further subcategories that can affect BGA. The other subcategories of “Past medical history” were genetic/congenital disorders, rheumatology/musculoskeletal diseases, and malignancy.

“Social status” data elements that affect BGA included opioid dependency, chronic alcohol consumption, sedative dependency, and tobacco chewing. The subcategories of “Physical examination” entailed vital signs, GCS, respiratory status, sedation status (RAS score), numeric pain scale, behavioral pain score, diaphoresis, shivering, cyanosis (if spO_2_ unavailable or suspicious), urine output, nasogastric drainage, edematous states, and poor tissue perfusion (regional hypo-perfusion). “Paraclinical investigation” category was all the examinations that can help analyze blood gas, including but not limited to hemoglobin, potassium, blood urea nitrogen, creatinine, chloride, glucose, lactate, anion and osmolar gap, as well as the related measurements. The complete proposed dataset for BGA is presented in Table 3.

**Table 3:** The complete proposed dataset for blood gas analysis

Number	Variable		Values		Yes (M + O) N (%)	No N (%)
**1-Personal Information, all are Mandatory**						
1–1	Medical Record number		Number		7 (87.50)	1 (12.50)
1–2	National code		Number		8 (100)	0 (0)
1–3	Age		Number		8 (100)	0 (0)
1–4	Sex		Male/Female/ Unknown		8 (100)	0 (0)
1–5	First name		Text		6 (75)	2 (25)
1–6	Last name		Text		6 (75)	2 (25)
1–7	Father's name		Text		6 (75)	2 (25)
1–8	Birth date		YYYY/MM/DD		6 (75)	2 (25)
1–9	Estimated height		Number		6 (75)	2 (25)
1–10	Estimated weight		Number		6 (75)	2 (25)
**2-Admission Details, all are Mandatory**						
2–1	Date of admission to hospital		YYYY/MM/DD		6 (75)	2 (25)
2–2	Time of admission to hospital		HH:MM		6 (75)	2 (25)
2–3	Date of admission to ICU		YYYY/MM/DD		6 (75)	2 (25)
2–4	Time of admission to ICU		HH:MM		6 (75)	2 (25)
2–5	Admission type		Medical 0 / Surgical 1		8 (100)	0 (0)
2–6	Surgical admission		Elective 0 / Emergency 1		8 (100)	0 (0)
2–7	Insurance coverage		No/Yes		6 (75)	2 (25)
2–8	Primary diagnosis		Text/Code		8 (100)	0 (0)
2–9	ICU diagnosis		Non-operative 0 / Post-operative 1		8 (100)	0 (0)
2–10	ICU intervention		Invasive ventilation 0		8 (100)	0 (0)
			Non-invasive ventilation 1			
			tracheostomy 2			
			ECMO 3			
			Renal replacement therapy 4			
			Inotropes/Vasopressor drug 5			
			Other 6			
			None 7			
**3-Present illness:**						
**3–1-Respiratory disease**						
3–1-1	Pneumonia		No/Yes	M	8 (100)	0 (0)
3–1-2	Pleural effusion		No/Yes	M	8 (100)	0 (0)
3–1-3	Pneumothorax		No/Yes	M	8 (100)	0 (0)
3–1-4	Profound hypoxemia		No/Yes	O	8 (100)	0 (0)
3–1-5	Respiratory aspiration		No/Yes	M	8 (100)	0 (0)
3–1-6	Hemothorax		No/Yes	M	8 (100)	0 (0)
3–1-7	Bronchitis		No/Yes	M	8 (100)	0 (0)
3–1-8	ARDS		No/Yes	M	8 (100)	0 (0)
3–1-9	Pulmonary Embolism		No/Yes	M	8 (100)	0 (0)
3–1-10	Post hypercapnic state		No/Yes	M	8 (100)	0 (0)
**3–2-Renal disease**						
3–2-1	Acute Kidney injury		No/Yes	M	8 (100)	0 (0)
3–2-2	Myoglobinuric acute renal failure		No/Yes	M	7 (87.50)	1 (12.50)
3–2-3	Uremia		No/Yes	M	7 (87.50)	1 (12.50)
3–2-4	Renal failure plus alkali therapy		No/Yes	O	6 (75)	2 (25)
3–2-5	Obstructive nephropathy		No/Yes	O	7 (87.50)	1 (12.50)
3–2-6	Renal transplant rejection		No/Yes	O	8 (100)	0 (0)
**3–3-Gastrointestinal disease/ Liver disease**						
3–3-1	Acute hepatic failure		No/Yes	M	8 (100)	0 (0)
3–3-2	Ischemic bowel		No/Yes	M	8 (100)	0 (0)
3–3-3	Small bowel obstruction		No/Yes	M	8 (100)	0 (0)
3–3-4	Diarrhea		No/Yes	M	8 (100)	0 (0)
3–3-5	Vomiting		No/Yes	M	8 (100)	0 (0)
3–3-6	Gastric aspiration		No/Yes	M	8 (100)	0 (0)
**3–4-Endocrine disease**						
3–4-1	Diabetic Ketoacidosis (acetoacetate)		No/Yes	M	8 (100)	0 (0)
3–4-2	Late stage in treatment of diabetic ketoacidosis		No/Yes	M	8 (100)	0 (0)
3–4-3	Hyperalbuminemia		No/Yes	O	8 (100)	0 (0)
3–4-4	Hypercalcemia- hypoparathyroidism		No/Yes	O	8 (100)	0 (0)
3–4-5	Cushing disease		No/Yes	O	6 (75)	2 (25)
3–4-6	Adrenal disease		No/Yes	O	8 (100)	0 (0)
3–4-7	Idiopathic hypercalciuria		No/Yes	O	6 (75)	2 (25)
**3–5-Cardiovascular disease**						
3–5-1	Shock		No 0	M	8 (100)	0 (0)
			Septic shock 1			
			Hypovolemic shock 2			
			Cardiogenic shock 3			
			Hemorrhagic shock 4			
			Obstructive shock 5			
			Other shock 6			
3–5-2	Accelerated hypertension		No/Yes	M	8 (100)	0 (0)
3–5-3	Cardiac failure		No/Yes	M	8 (100)	0 (0)
**3–6-Hematologic disease**						
3–6-1	Paroxysmal nocturnal hemoglobinuria		No/Yes	O	6 (75)	2 (25)
3–6-2	Hyperglobulinemic purpura		No/Yes	O	6 (75)	2 (25)
**3–7-Neurologic disease**						
3–7-1	Active seizure		No/Yes	M	8 (100)	0 (0)
3–7-2	Recent CVA		No/Yes	M	8 (100)	0 (0)
3–7-3	CNS infections		No/Yes	M	8 (100)	0 (0)
3–7-4	Encephalitis		No/Yes	M	8 (100)	0 (0)
3–7-5	Meningitis		No/Yes	M	8 (100)	0 (0)
3–7-6	Muscular dystrophy		No/Yes	O	7 (87.50)	1 (12.50)
**3–8-Infectious disease**						
3–8-1	Sepsis		No/Yes	M	8 (100)	0 (0)
3–8-2	Cholera		No/Yes	O	6 (75)	2 (25)
3–8-3	Acute Poliomyelitis		No/Yes	O	7 (87.50)	1 (12.50)
**3–9-Trauma**						
3–9-1	Heat exposure		No/Yes	O	8 (100)	0 (0)
3–9-2	High altitude		No/Yes	O	8 (100)	0 (0)
3–9-3	Barotrauma		No/Yes	O	8 (100)	0 (0)
3–9-4	Acute starvation		No/Yes	M	8 (100)	0 (0)
3–9-5	Rhabdomyolysis		No/Yes	M	7 (87.50)	1 (12.50)
3–9-6	Severe trauma		No/Yes	M	8 (100)	0 (0)
**3–10-Drugs**						
3–10-1	Diuretics		No 0	M	8 (100)	0 (0)
			Thiazide 1			
			Acetazolamide 2			
			Furosemide 3			
			Triamterene 4			
			Spironolactone 5			
			Other 6			
3–10-2	Calcium chloride		No/Yes	O	8 (100)	0 (0)
3–10-3	Magnesium sulfate		No/Yes	M	8 (100)	0 (0)
3–10-4	Cholestyramine		No/Yes	O	8 (100)	0 (0)
3–10-5	Paraldehyde		No/Yes	B	8 (100)	0 (0)
3–10-6	Sorbitol		No/Yes	M	8 (100)	0 (0)
3–10-7	Angiotensin-converting enzyme inhibitors (ACE inhibitors)		No/Yes	M	7 (87.50)	1 (12.50)
3–10-8	angiotensin 2 receptor blockers (ARBs)		No/Yes	M	7 (87.50)	1 (12.50)
3–10-9	Digoxin /digitalis		No/Yes	M	7 (87.50)	1 (12.50)
3–10-10	Beta adrenergic antagonist		No/Yes	M	7 (87.50)	1 (12.50)
3–10-11	α adrenergic agonists		No/Yes	M	7 (87.50)	1 (12.50)
3–10-12	Somatostatine		No/Yes	M	7 (87.50)	1 (12.50)
3–10-13	Diazoxide		No/Yes	O	7 (87.50)	1 (12.50)
3–10-15	Arginine hydrochloride		No/Yes	O	7 (87.50)	1 (12.50)
3–10-16	Lysine hydrochloride		No/Yes	O	5 (62.50)	3 (37.50)
3–10-17	Acute alkali administration		No/Yes	O	8 (100)	0 (0)
3–10-18	Kayeoxalate		No/Yes	M	8 (100)	0 (0)
3–10-19	Fludrocortisone		No/Yes	O	8 (100)	0 (0)
3–10-20	Combined administration of sodium polystyrene sulfonate (kayexalate and aluminum hydroxide)		No/Yes	M	8 (100)	0 (0)
3–10-21	Penicillin (Non reabsorbable anions)		No/Yes	M	7 (87.50)	1 (12.50)
3–10-22	Carbenicillin (Non reabsorbable anions)		No/Yes	M	8 (100)	0 (0)
3–10-23	Bumetanide		No/Yes	M	6 (75)	2 (25)
3–10-24	Nonsteroidal anti-inflammatory drugs (NSAIDs)		No/Yes	M	7 (87.50)	1 (12.50)
3–10-25	Cyclosporine		No/Yes	M	8 (100)	0 (0)
3–10-26	IV xylose		No/Yes	O	6 (75)	2 (25)
3–10-27	IV sorbitol		No/Yes	O	7 (87.50)	1 (12.50)
3–10-28	Ethanol		No/Yes	M	8 (100)	0 (0)
3–10-29	Ifosfamide		No/Yes	O	6 (75)	2 (25)
3–10-30	Amphotericin B		No/Yes	M	8 (100)	0 (0)
3–10-31	Foscarnet		No/Yes	O	7 (87.50)	1 (12.50)
3–10-32	Streptozotocin		No/Yes	O	6 (75)	2 (25)
3–10-33	Amiloride		No/Yes	O	6 (75)	2 (25)
3–10-35	Trimethoprim		No/Yes	M	7 (87.50)	1 (12.50)
3–10-36	Tacrolimus		No/Yes	M	7 (87.50)	1 (12.50)
3–10-37	Intravenous (IV) fructose		No/Yes	O	7 (87.50)	1 (12.50)
3–10-38	Methenamin Hippurate		No/Yes	O	6 (75)	2 (25)
3–10-39	Ammonium chloride		No/Yes	O	7 (87.50)	1 (12.50)
3–10-40	Total parental nutrition (TPN)		No/Yes	M	8 (100)	0 (0)
3–10-41	rapid saline administration		No/Yes	M	8 (100)	0 (0)
3–10-42	Nonnucleoside antireverse transcriptase drugs		No/Yes	M	7 (87.50)	1 (12.50)
3–10-43	sulfanilamide		No/Yes	O	7 (87.50)	1 (12.50)
3–10-44	Mafenide acetate		No/Yes	B	7 (87.50)	1 (12.50)
3–10-45	Lithium		No/Yes	M	7 (87.50)	1 (12.50)
3–10-46	Heparin (low MW or unfractionated) in critical ill patients		No/Yes	M	7 (87.50)	1 (12.50)
3–10-47	Carbenoxolone		No/Yes	O	6 (75)	2 (25)
3–10-48	Estrogen		No/Yes	M	7 (87.50)	1 (12.50)
3–10-49	Bicarbonate therapy of organic acidosis		No/Yes	M	8 (100)	0 (0)
3–10-50	Morphine		No/Yes	M	7 (87.50)	1 (12.50)
3–10-51	Sedative		No/Yes	M	7 (87.50)	1 (12.50)
3–10-52	Renin angiotensin system modulating agents(ACEI, ARB)		No/Yes	M	5 (62.50)	3 (37.5)
3–10-53	Mannitol		No/Yes	M	8 (100)	0 (0)
3–10-54	Metformin		No/Yes	M	8 (100)	0 (0)
3–10-55	Glucocorticoid		No/Yes	M	6 (75)	2 (25)
3–10-56	Ectopic corticotropin		No/Yes	O	6 (75)	2 (25)
**3–11-Toxins**						
3–11-1	Methanol		No/Yes	M	8 (100)	0 (0)
3–11-2	Ethanol		No/Yes	M	8 (100)	0 (0)
3–11-3	Ethylene glycol		No/Yes	O	8 (100)	0 (0)
3–11-4	Propylene glycol		No/Yes	O	7 (87.50)	1 (12.50)
3–11-5	Isopropyl alcohol poisoning		No/Yes	O	7 (87.50)	1 (12.50)
3–11-6	Acetone poisoning		No/Yes	O	7 (87.50)	1 (12.50)
3–11-7	Methyl alcohol		No/Yes	O	7 (87.50)	1 (12.50)
3–11-8	Salicylate intoxication		No/Yes	M	8 (100)	0 (0)
3–11-9	Paraldehyde		No/Yes	O	8 (100)	0 (0)
3–11-10	Toluene		No/Yes	O	7 (87.50)	1 (12.50)
3–11-11	Pyroglutamic (5-oxoproline)		No/Yes	O	7 (87.50)	1 (12.50)
3–11-12	Cyanide		No/Yes	O	7 (87.50)	1 (12.50)
3–11-13	2,4 dinitrophenol		No/Yes	O	6 (75)	2 (25)
3–11-14	Carbon monoxide poisoning		No/Yes	O	8 (100)	0 (0)
3–11-15	Lead		No/Yes	M	7 (87.50)	1 (12.50)
3–11-16	Vitamin D toxicity		No/Yes	O	6 (75)	2 (25)
3–11-17	Outdated Tetracycline		No/Yes	O	6 (75)	2 (25)
3–11-18	Sulfur ingestion		No/Yes	O	6 (75)	2 (25)
3–11-19	NH4CI ingestion		No/Yes	O	6 (75)	2 (25)
3–11-20	Alcohols metabolized by alcohol dehydrogenase		No/Yes	M	6 (75)	2 (25)
3–11-21	Methicillin nephrotoxicity		No/Yes	O	6 (75)	2 (25)
**4-Past Medical History:**						
**4–1-Respiratory disease**						
4–1-1	COPD		No/Yes	M	7 (87.50)	1 (12.50)
4–1-2	Asthma (severe)		No/Yes	M	8 (100)	0 (0)
4–1-3	Other obstructive lung disease		No/Yes	O	8 (100)	0 (0)
4–1-4	Sleep disorder breathing (OSA or OHS)		No/Yes	M	8 (100)	0 (0)
4–1-5	Pleural effusion		No/Yes	M	8 (100)	0 (0)
4–1-6	Pneumoconiosis		No/Yes	O	8 (100)	0 (0)
4–1-7	Emphysema		No/Yes	M	8 (100)	0 (0)
4–1-8	Cystic fibrosis		No/Yes	O	8 (100)	0 (0)
4–1-9	Fibrosing alveolitis		No/Yes	O	8 (100)	0 (0)
**4–2-Renal disease**						
4–2-1	Chronic kidney disease		No/Yes	M	8 (100)	0 (0)
4–2-2	ESRD		No/Yes	M	8 (100)	0 (0)
4–2-3	Nephrosclerosis		No/Yes	O	6 (75)	2 (25)
4–2-4	Bartter syndrome		No/Yes	O	7 (87.50)	1 (12.50)
4–2-5	Gitelmans syndrome		No/Yes	O	7 (87.50)	1 (12.50)
4–2-6	Renal artery stenosis		No/Yes	O	7 (87.50)	1 (12.50)
4–2-7	Liddles syndrome		No/Yes	O	7 (87.50)	1 (12.50)
4–2-8	Balkan nephropathy		No/Yes	O	7 (87.50)	1 (12.50)
4–2-9	Nephrocalcinosis		No/Yes	O	6 (75)	2 (25)
4–2-10	HIV nephropathy		No/Yes	O	5 (62.50)	3 (37.50)
4–2-11	Chronic pyelonephritis		No/Yes	M	7 (87.50)	1 (12.50)
4–2-12	Medullary cystic disease		No/Yes	O	6 (75)	2 (25)
4–2-13	Renal transplantation		No/Yes	M	8 (100)	0 (0)
4–2-14	Nephrotic syndrome		No/Yes	M	8 (100)	0 (0)
4–2-15	Diabetic nephropathy		No/Yes	M	8 (100)	0 (0)
4–2-16	Tubulointerstitial nephropathies		No/Yes	M	5 (62.50)	3 (37.50)
4–2-17	Lupus nephritis		No/Yes	O	7 (87.50)	1 (12.50)
4–2-18	Obstructive nephropathy		No/Yes	M	7 (87.50)	1 (12.50)
**4–3-Gastrointestinal disease/ Liver disease**						
4–3-1	Chronic liver failure/Cirrhosis		No/Yes	M	8 (100)	0 (0)
4–3-2	Short bowel syndrome		No/Yes	O	8 (100)	0 (0)
4–3-3	Fistula, Enteral/external (external pancreatic or small bowel drainage, uterosigmoidostomy, jejunal loop)		No/Yes	O	8 (100)	0 (0)
4–3-4	Villous adenoma		No/Yes	O	5 (62.50)	3 (37.50)
4–3-5	Ileostomy		No/Yes	M	5 (62.50)	3 (37.50)
4–3-6	Jejunoileal bypass with hyperoxaluria		No/Yes	O	8 (100)	0 (0)
4–3-7	Jejunal bypass with hyperoxaluria		No/Yes	O	7 (87.50)	1 (12.50)
**4–4-Endocrine disease**						
4–4-1	Hyperthyroidism		No/Yes	M	8 (100)	0 (0)
4–4-2	Diabetes mellitus		No/Yes	M	8 (100)	0 (0)
4–4-3	Pheochromocytoma		No/Yes	O	8 (100)	0 (0)
4–4-4	Addison disease		No/Yes	O	8 (100)	0 (0)
4–4-5	Bilateral adrenalectomy		No/Yes	O	8 (100)	0 (0)
4–4-6	Hypercalcemia- hypoparathyroidism		No/Yes	M	8 (100)	0 (0)
4–4-7	Milk – alkali syndrome		No/Yes	O	8 (100)	0 (0)
4–4-8	Renin secreting tumor		No/Yes	O	8 (100)	0 (0)
4–4-9	Primary aldosteronism		No/Yes	O	8 (100)	0 (0)
4–4-10	Familial hypoaldosteronism		No/Yes	O	7 (87.50)	1 (12.50)
4–4-11	Chronic idiopathic hypoaldosteronism		No/Yes	O	7 (87.50)	1 (12.50)
4–4-12	Desmolase deficiency		No/Yes	O	7 (87.50)	1 (12.50)
4–4-13	Adrenal carcinoma/adenoma		No/Yes	O	8 (100)	0 (0)
4–4-14	Adrenal hyperplasia		No/Yes	O	7 (87.50)	1 (12.50)
4–4-15	Adrenal destruction		No/Yes	O	8 (100)	0 (0)
4–4-16	Primary pituitary adenoma		No/Yes	O	6 (75)	2 (25)
4–4-17	Conn’s syndrome		No/Yes	O	5 (62.50)	3 (37.50)
4–4-18	Idiopathic hypercalciuria		No/Yes	O	5 (62.50)	3 (37.50)
4–4-19	Primary hyperparathyroidism		No/Yes	O	7 (87.50)	1 (12.50)
4–4-20	Secondary hyperparathyroidism with chronic hypocalcemia:Vitamin D deficiency or resistance		No/Yes	O	8 (100)	0 (0)
4–4-21	Secondary hyperparathyroidism with chronic hypocalcemia:Vitamin D dependency		No/Yes	O	8 (100)	0 (0)
4–4-22	Hyperaldosteronism		No/Yes	O	7 (87.50)	1 (12.50)
4–4-23	Hyperreninemia		No/Yes	O	6 (75)	2 (25)
4–4-24	Obesity		No/Yes	O	5 (62.50)	3 (37.50)
**4–5-Cardiovascular disease**						
4–5-1	Cardiac failure		No/Yes	M	8 (100)	0 (0)
**4–6-Hematologic disease**						
4–6-1	Hereditary elliptocytosis		No/Yes	O	7 (87.50)	1 (12.50)
4–6-2	Sickle cell anemia		No/Yes	O	8 (100)	0 (0)
4–6-3	Multiple myeloma		No/Yes	O	8 (100)	0 (0)
4–6-4	Profound anemia		No/Yes	M	8 (100)	0 (0)
4–6-5	Paroxysmal nocturnal hemoglobinouria		No/Yes	O	6 (75)	2 (25)
**4–7-Neurologic disease**						
4–7-1	CVA		No/Yes	M	8 (100)	0 (0)
4–7-2	CNS tumors		No/Yes	M	8 (100)	0 (0)
4–7-3	Myasthenia gravis		No/Yes	M	8 (100)	0 (0)
4–7-4	Multiple Schlerosis		No/Yes	M	8 (100)	0 (0)
4–7-5	Neuroblastoma		No/Yes	O	6 (75)	2 (25)
**4–8-Genetic/Congenital disorders**						
4–8-1	Congenital chloridorrhea		No/Yes	O	7 (87.50)	1 (12.50)
4–8-2	Fanconi syndrome		No/Yes	O	5 (62.50)	3 (37.50)
4–8-3	Pseudohypoaldosteronism-1 (PHA-1)		No 0	O	6 (75)	2 (25)
			autosomal dominant 1			
			Autosomal recessive 2			
4–8-4	Pseudohypoaldosteronism-2 (PHA-2)		No	O	6 (75)	2 (25)
			Autosomal dominant 1			
4–8-5	Inborn errors of metabolism		No/Yes	O	8 (100)	0 (0)
4–8-6	Coricosterone methyloxidase deficiency		No 0	O	6 (75)	2 (25)
			type I 1			
			Type II 2			
4–8-7	Primary zona glomerulosa defect		No/Yes	O	6 (75)	2 (25)
4–8-8	Transient hypoaldosteronism of infancy		No/Yes	O	6 (75)	2 (25)
4–8-9	Galactosemia		No/Yes	O	7 (87.50)	1 (12.50)
4–8-10	Hereditary fructose intolerance		No/Yes	O	7 (87.50)	1 (12.50)
4–8-11	Metachromatic leukodystrophy		No/Yes	O	6 (75)	2 (25)
4–8-12	Pyruvate carboxylase deficiency		No/Yes	O	7 (87.50)	1 (12.50)
4–8-13	Methylmalonic acidemia		No/Yes	O	7 (87.50)	1 (12.50)
4–8-14	Fabry disease		No/Yes	O	6 (75)	2 (25)
4–8-15	Carnitine palmitoyltransferase		No/Yes	O	6 (75)	2 (25)
4–8-16	Carbonic anhydrase 2 deficiency with osteopetrosis (Sly syndrome)		No/Yes	O	6 (75)	2 (25)
4–8-17	21 hydroxylase deficiency		No/Yes	O	6 (75)	2 (25)
4–8-18	3 beta –hydroxydehrogenase deficiency		No/Yes	O	6 (75)	2 (25)
4–8-19	Hereditary (Congenital) sensorineural deafness		No/Yes	O	6 (75)	2 (25)
4–8-20	Carbonic anhydrase deficiency or inhibition		No/Yes	O	6 (75)	2 (25)
4–8-21	Tyrosinemia		No/Yes	O	6 (75)	2 (25)
**4–9-Infectious disease**						
4–9-1	Acquired immunodeficiency syndrome		No/Yes	O	5 (62.50)	3 (37.50)
**4–10-Rheumatology/musculoskeletal disease**						
4–10-1	polyarteritis nodosa		No/Yes	O	7 (87.50)	1 (12.50)
4–10-2	Sjögren's syndrome		No/Yes	O	7 (87.50)	1 (12.50)
4–10-3	Kyphoscoliosis		No/Yes	O	7 (87.50)	1 (12.50)
4–10-4	Muscular dystrophies		No/Yes	O	7 (87.50)	1 (12.50)
**4–11- Malignancy (b)**					8 (100)	0 (0)
**5-Societal status**						
5–1	Opioid dependency		No/Yes	M	8 (100)	0 (0)
5–2	Chronic alcohol use		No/Yes	M	8 (100)	0 (0)
5–3	Sedatives dependency		No/Yes	M	8 (100)	0 (0)
5–4	Tobacco chewer		No/Yes	M	6 (75)	2 (25)
**6-Physical examination**						
**6–1-Vital signs**						
6–1-1		Last body temperature		M	8 (100)	0 (0)
6–1-2		Last systolic blood pressure		M	8 (100)	0 (0)
6–1-3		Last diastolic blood pressure		M	8 (100)	0 (0)
6–1-4		Last MAP (mean arterial pressure)		M	8 (100)	0 (0)
6–1-5		Heart rate (beat per minute)		M	8 (100)	0 (0)
6–1-6		Last total respiratory rate		M	8 (100)	0 (0)
6–1-7		Last spO2		M	8 (100)	0 (0)
**6–2-GCS (physician note)**					8 (100)	0 (0)
**6–3-Respiratory:**						
6–3-1		Spontaneous breathing (FiO2%)		M	8 (100)	0 (0)
6–3-2		Assisted (FiO2%)		M	8 (100)	0 (0)
6–3-3		PaO2/FiO2 Ratio		M	8 (100)	0 (0)
6–3-4		Flail chest (Yes/No)		M	8 (100)	0 (0)
**6–4- sedation status (RAS score)**				M	7 (87.50)	1 (12.50)
**6–5-Numeric pain scale**				M	8 (100)	0 (0)
**6–6-Behavioral Pain Score**				M	7 (87.50)	1 (12.50)
**6–7-Diaphoresis**(Yes/No)				M	8 (100)	0 (0)
**6–8-Shivering**(Yes/No)				M	7 (87.50)	1 (12.50)
**6–9-Cyanosis (if spO2 unavailable or suspicious)** (Yes/No)				M	8 (100)	0 (0)
**6–10-Urine output**				M	8 (100)	0 (0)
**6–11-Nasogastric drainage**				M	8 (100)	0 (0)
**6–12-Edematous states**(Yes/No)				M	8 (100)	0 (0)
**6–13-Poor tissue perfusion (regional hypo-perfusion)** (Yes/No)				M	8 (100)	0 (0)
**7-Para-clinical investigation**						
7–1		Last WBC		M	7 (87.50)	1 (12.50)
7–2		Last Hb		M	8 (100)	0 (0)
7–3		Last MetHb		O	7 (87.50)	1 (12.50)
7–4		Last CarboxyHb		O	7 (87.50)	1 (12.50)
7–5		Last plt		M	6 (75)	2 (25)
7–6		Last Na		M	8 (100)	0 (0)
7–7		Last K		M	8 (100)	0 (0)
7–8		Last BUN		M	8 (100)	0 (0)
7–9		Last Creatinin		M	8 (100)	0 (0)
7–10		Urine for oxalate crystals (ethylene glycol)		O	8 (100)	0 (0)
7–11		Last Mg		O	8 (100)	0 (0)
7–12		Last Ca		O	8 (100)	0 (0)
7–13		Last Cl		O	8 (100)	0 (0)
7–14		Last Glucose		M	8 (100)	0 (0)
7–15		Last Lactate		O	8 (100)	0 (0)
7–16		Last Bilirubin		O	6 (75)	2 (25)
7–17		Last Albumin		O	8 (100)	0 (0)
7–18		Last Li^+^		O	7 (87.50)	1 (12.50)
7–19		Last sulfate		O	6 (75)	2 (25)
7–20		Last phosphate		O	8 (100)	0 (0)
7–21		Last IgG		O	8 (100)	0 (0)
7–22		Hyperviscosity		O	6 (75)	2 (25)
7–24		Thiamine (B1) level		O	6 (62.50)	3 (37.50)
7–25		Anion Gap:		M	8 (100)	0 (0)
7–26		Corrected Anion Gap		M	8 (100)	0 (0)
7–27		Albumin Gap		M	8 (100)	0 (0)
7–28		Osmolality measured		O	8 (100)	0 (0)
7–29		Osmolality calculated		O	8 (100)	0 (0)
7–30		Osmolar gap		O	8 (100)	0 (0)
**8-Blood gas parameter**						
8–1		Last ABG		M	8 (100)	0 (0)
8–2		pH		M	8 (100)	0 (0)
8–3		pCO2		M	8 (100)	0 (0)
8–4		pO2		M	8 (100)	0 (0)
8–5		O2 saturation		M	8 (100)	0 (0)
8–6		HCO3		M	8 (100)	0 (0)
8–7		Base Excess/base deficit		M	8 (100)	0 (0)
**9-SOFA score, all are mandatory**						
9–1		PaO2/FiO2 (mmHg)			8 (100)	0 (0)
9–2		GCS			8 (100)	0 (0)
9–3		Mean arterial pressure (MAP)			8 (100)	0 (0)
9–4		Bilirubin (mg/dl) [μmol/L]			7 (87.50)	1 (12.50)
9–5		Platelets × 103/ml			6 (75)	2 (25)
9–6		Creatinine (mg/dl) [μmol/L]			8 (100)	0 (0)
**10-Sampling technique (ABG Error), all are mandatory**						
10–1		Steady state	No/Yes		8 (100)	0 (0)
10–2		Anticoagulants (Excess)	No/Yes		8 (100)	0 (0)
10–3		Processing delay	Number (Minute)		8 (100)	0 (0)
10–4		Venous sampling:	No/Yes		8 (100)	0 (0)
10–5		Acceptable pulse oximetry care	No/Yes		8 (100)	0 (0)
10–6		SpO2 calculated by pulse oximetry	No/Yes		8 (100)	0 (0)
10–7		Sampling equipment	Dead space: … (volume)		8 (100)	0 (0)
			Needle gauge ≥ 25:			
			No 0			
			Yes 1			
			Needle size:			
			25			
			35			
10–8		Ventilator status	Mechanical ventilation 0		8 (100)	0 (0)
			Non mechanical ventilation 1			
10–9		Mode of ventilation and information on oxygen supply	According to admission/progress note		8 (100)	0 (0)
10–10		Request for related measurements (electrolytes, metabolites)	yes/no		8 (100)	0 (0)
10–11		Person collecting the sample	Novice		8 (100)	0 (0)
			Experienced			
10–12		CO Oximetry	No/Yes		8 (100)	0 (0)

*M* mandatory data element, *O* optional data element, *N* Number, *YYYY/MM.DD* year with four digits, month with two digits, day with two digits, *HH:MM* hour with two digits and minute with two digits, *ECMO* Extracorporeal membrane oxygenation, *ARDS* Acute Respiratory Distress Syndrome, *CVA* Cerebrovascular Accident, *CNS* Central Nervous System, *COPD* Chronic Obstructive Pulmonary Disease, *PAC* plasma aldosterone concentration, *PRA* plasma renin activity, *PRC* plasma renin concentration, *SOFA* The Sequential Organ Failure Assessment Score, *GCS* Glasgow Coma Scale, *CO* Carbon monoxide

## Discussions

In the present study, 313 data elements were approved by the experts to be contained in the dataset, including 172 mandatory and 141 optional data elements. These data elements were categorized into ten main categories, namely “Personal information”, “Admission details”, “Present illnesses”, “Past medical history”, “Social status”, “Physical examination”, “Paraclinical investigation”, “Blood gas parameters”, “SOFA score”, and “Sampling technique errors (ABG Error)”.

Despite the wide adoption of AI-based applications, such as machine learning in ICUs, to our knowledge, this is the first developed dataset of data elements required for comprehensive BGA. However, according to the systematic reviews performed by Syed et al. and Shillan et al. [31, 32], machine learning applications are widely applied for predicting ICU mortality, readmission, acute kidney injury, and sepsis. Although advances in AI-bassed techniques have turned from “a future possibility” to an “everyday reality” for managing patients in ICUs, there are still challenges in the usage of these systems [33].

Due to the lack of interoperability of electronic systems which results in a lack of data integration, the potential of hospital data for solving healthcare problems is yet to be fully realized. Developing AI-based systems requires large datasets for modeling complex and non-linear effects or developing evidence-based algorithms [34, 35]. In an attempt to cover this issue in intensive care, Johnson et al. [25] released the Medical Information Mart for Intensive Care (MIMIC-III) dataset that allows researchers to solve complex healthcare problems through developing electronic systems [31]. For instance, through extracting relevant features from the MIMIC-III dataset, Yang et al. [36] proposed an algorithm based on the non-invasive physiological parameters of patients to calculate the partial pressure of oxygen/fraction of inspired oxygen (PaO_2_/FiO_2_) ratio for the identification of patients with acute respiratory distress syndrome. However, contrary to our proposed dataset, the MIMIC-III dataset does not contain all the specific data required for BGA. Our proposed dataset has the potential to be used as a base for developing such databases.

Some of the obtained data elements in our study are similar to those of previous investigations. Australian and New Zealand Intensive Care Society (ANZICS) has built one of the largest single datasets for ICU adult patients [26]. It contains a section named “blood gases” which collects data on the date and time of blood gas test, FiO_2_, PaO_2_, the partial pressure of carbon dioxide (PaCO_2_), pH, and whether patients were intubated. However, it lacks many of the data elements required for automatic BGA. In addition to these essential data elements, our dataset contains diseases, drugs, toxins, and other paraclinical investigations which might affect blood gas interpretation. As a secondary verification or rather a confirmation practice, we recommend further evaluations of AI methods, such as machine learning using the proposed dataset in future studies.

One concern in the proposed dataset is the high number of data elements required for automatic BGA. Many of these data elements can be uploaded using the existing electronic systems. For instance, a dataset has been developed for collecting progress notes data in Nemazee hospital [37]. It helped the electronic documentation of progress notes in the ICU. Therefore, it can be used to feed AI-based decision support systems designed for BGA. Another solution is a parent–child format of the dataset. The main category of “Past medical history” is a parent with eleven children. The AI-based decision support system requires the users to answer to a parent (with “YES” or “NO”). If “NO” is selected none of the children will be shown, and the system would ask the user to answer to the next parent, for example, “social status” with “YES” or “NO”. This approach would prevent designing a primitive user interface with complex menus and lots of scrolling to fill out the required data elements, which are not suited to the fast pace of the ICUs. Through reviewing the trend of “monitoring” and “data acquisition” systems in ICUs, Georgia et al. [38] found that acquiring, synchronizing, integrating, and analyzing patient data is difficult because of the insufficient computational power and a lack of specialized software, incompatibility between monitoring equipment, and limited data storage. The development and application of datasets in practice assist in removing these technical challenges. Moreover, creating “mandatory” or “optional” divisions allows decreasing the data elements to save the time required for BGA, which means if the user selects a simple analysis, the data elements, required to be filled, will dramatically decrease.

## Conclusion

We proposed a dataset as a base for developing AI-based systems to assist BGA. It helps the storage of accurate and comprehensive data, as well as the integration of these data in other information systems. Moreover, it contributes to the provision of high-quality care and better clinical decision-making through implementing the AI methods that help manage patients. This dataset has the potential to foster building databases with ICUs which is helpful for researchers, students, and policymakers for improving patients care in ICUs.

## Data Availability

All data are presented in the manuscript submission.

## Abbreviations

AI: Artificial intelligence
BGA: Blood Gas Analysis
ICU: Intensive Care Unit
SOFA: Sequential Organ Failure Assessment
PaCO2: Partial Pressure Of Carbon Dioxide
EHR: Electronic Health Record
CPOE: Computerized Physician Order Entry
ABG Error: Arterial Blood Gas Sampling Technique Errors
ANZICS: Australian and New Zealand Intensive Care Society
